# Supplementary material for: Soluble Neuropilin-1 as a Marker for Distinguishing Bacterial and Viral Sepsis in Critically Ill Patients—A Prospective, Multicenter, Observational Study
Source: Viruses. 2025 Jul 16;17(7):997. doi: 10.3390/v17070997 (PMC12297973; doi:10.3390/v17070997)
Supplement: Supplementary file 1 [file viruses-17-00997-s001.zip › viruses-3761119-supplementary.pdf]

Electronic supplemental material

Soluble Neuropilin-1 as a marker for distinguishing bacterial and viral sepsis in critically ill patients – a prospective, multicentre, observational study

Fabian Perschinka[1], Georg Franz Lehner[1], Timo Mayerhöfer1, Frank Hartig[1], Birgit Zassler[1], Johannes Bösch[2], Dietmar Fries[2], Romuald Bellmann[1], Michael Joannidis[1]

ESM Table S1: Diagnosed pathogens at ICU admission suspected causing the sepsis

Bacterial	Viral
1x Actinomyces naeslundii	1x Enterovirus
1x Areomonas caviae	4x Influenza A
1x Clamydophila pneumoniae	9x SARS-CoV-2
1x Enterobacter cloacae-complex	
1x Enterococcus malodoratus	
2x Enterococcus faecium	
7x Escherichia coli	
1x Haemophilus influenzae	
1x Klebsiella oxytoca	
4 x Klebsiella pneumoniae	
1x Klebsiella variicola	
2x Legionella pneumophila	
2x Pseudomonas aeruginosa	
1x Providencia rettgeri	
4x Staphylococcus aureus	
1x Staphylococcus hominis	
1x Streptococcus anginosus	
1x Streptococcus dysgalactiae	
1x Streptococcus gordonii	
4x Streptococcus pneumoniae	
3x Streptococcus pyogenes	

All pathogens were listed in case of multiple detected pathogens

ESM Table S2: Comparison of interventions and medication in bacterial and viral sepsis patients

	Bacterial (n = 37)	Viral (n = 14)	p
IMV*	21 (56.8%)	12 (85.7%)	0.053
Vasopressors*	37 (100.0%)	12 (85.7%)	0.019
AKI*	28 (77.8%)	8 (57.1%)	0.145
KDIGO stage 1*	4 (14.3%)	1 (12.5%)	
KDIGO stage 2*	8 (28.6%)	3 (37.5%)	0.890
KDIGO stage 3*	16 (57.1%)	4 (50.0%)	
RRT*	14 (37.8%)	5 (35.7%)	0.889
ECMO*	1 (2.7%)	1 (7.1%)	0.466
IMV days°	11 (6 – 20)	12 (6 – 32)	0.699
NIV days°	2 (1 – 4)	4 (2 – 6)	0.446
NHF days°	3 (1 – 4)	3 (2 – 7)	0.227
RRT days°	7 (4 – 16)	7 (4 – 19)	0.754
ECMO days°	11 (11 – 11)	5 (5 – 5)	1.000
Use of antibiotics	37 (100.0%)	14 (100.0%)	-
Use of antivral drugs	0	5 (35.7%)	< 0.001
Methylprednisolone*	2 (5.4%)	2 (14.3%)	0.292
Dexamethasone*	1 (2.7%)	7 (50.0%)	< 0.001
Hydrocortisone*	30 (81.1%)	3 (21.4%)	< 0.001
Other corticosteroids*	7 (18.9%)	0	0.080
No corticosteroid administration*	7 (18.9%)	1 (7.1%)	0.320
Methylprednisolone days°	10 (1 – 19)	7 (5 – 8)	1.000
Dexamethasone days°	1 (1 – 1)	10 (10 – 20)	0.250
Hydrocortisone days°	5 (4 – 9)	3 (2 – 7)	0.168
Other corticosteroid days°	3 (1 – 6)	-	-
Cumulative dose of Methylprednisolone (mg)°	662 (300 – 1024)	820 (320 – 1320)	1.000
Cumulative dose of Dexamethasone (mg)°	13 (13 – 13)	60 (60 – 198)	0.250
Cumulative dose of Hydrocortisone (mg)°	776 (450 – 1250)	250 (200 – 1250)	0.211

* n (%); ° median (IQR)

IMV: invasive mechanical ventilation; AKI: acute kidney injury; KDIGO: Kidney Disease: Improving Global Outcomes; RRT: renal replacement therapy; ECMO: extracorporeal membrane oxygenation; NIV: non-invasive ventilation; NHF: nasal high flow; mg: milligram.

ESM Table S3: Comparison of the detection of coinfections per day in the viral and the bacterial sepsis group

	Day after study inclusion	Pathogen	Specimen	Time to positivity (if applicable)
Viral sepsis group	Day 1	Cutibacterium acnes	Blood culture	107.16h
		Staphylococcus epidermidis	Blood culture	13.89h
		Bacillus pumilus	Blood culture	37.03h
		Staphylococcus aureus	Nasal Swab and	-
			Oropharyngeal Swab	-
	Day 2			
	Day 3			
	Day 4			
	Day 5	Yeast fungus	Bronchial secretion colonization	-
		Yeast fungus	Bronchial secretion colonization	-
	Day 6			
	Day 7			
Bacterial sepsis group	Day 1	Yeast fungus	Bronchial secretion colonization	-
		Yeast fungus	Bronchial secretion colonization	-
		Yeast fungus	Bronchial secretion colonization	-
	Day 2	Candida albicans	Blood culture	38.12h
		Escherichia coli	Blood culture	12.23h
		Yeast fungus	Bronchial secretion colonization	-
	Day 3			
	Day 4	Yeast fungus	Bronchial secretion colonization	-
	Day 5	Yeast fungus	Bronchial secretion colonization	-
	Day 6			
	Day 7	Enterococcus faecium	Oropharyngeal Swab	-
		Yeast fungus	Bronchial secretion colonization	-

ESM Table S4: Area under the ROC curve (AUC) values of inflammatory parameters for detecting bacterial infection

	IL-6	PCT	CRP
Day 1	0.859	0.836	0.712
Day 2	0.798	0.893	0.792
Day 3	0.716	0.882	0.763
Day 4	0.621	0.861	0.726
Day 5	0.429	0.902	0.636
Day 6	0.559	0.929	0.525
Day 7	0.406	0.932	0.460

ESM Table S5: Comparison of the baseline characteristics restricted to patients with a respiratory focus

	Bacterial (n = 17)	Viral (n = 14)	p
Age°	56 (50 – 66)	65 (57 – 71)	0.297
Sex (male)*	13 (76.5%)	11 (78.6%)	0.889
BMI°	24.8 (20.3 – 26.9)	26.2 (23.2 – 28.4)	0.385
HbA1c%°	5.8 (5.6 – 6.0)	6.0 (5.9 – 6.5)	0.241
SOFA score°	8 (7 – 10)	7 (6 – 8)	0.017
SAPS III°	69 (61 – 76)	61 (46 – 65)	0.033
Vasopressor administration at admission*	16 (94.1%)	11 (78.5%)	0.398
Norepinephrin at admission (µg/kg/min)°	0.17 (0.06 – 0.36)	0.17 (0.08 – 0.21)	0.716
Vasopressin at admission (U/hr)°	1.60 (1.60 – 1.60)	0.8 (0.8 – 0.8)	0.500
**Inflammatory markers at ICU admission**			
Interleukin-6 (ng/l)°	2513.0 (1678.0 – 4833.0)	145.0 (68.4 – 190.0)	< 0.001
C-reactive Protein (mg/dl)°	19.0 (7.4 – 32.2)	10.6 (4.6 – 19.5)	0.128
Procalcitonin (µg/l)°	12.3 (2.8 – 26.8)	0.3 (0.2 – 0.6)	< 0.001
**Comorbidities**			
Hypertension*	6 (35.3%)	7 (50.0%)	0.409
Coronary artery disease*	5 (29.4%)	6 (42.9%)	0.436
Atrial fibrillation*	5 (29.4%)	3 (21.4%)	0.613
COPD*	7 (41.2%)	1 (7.1%)	0.031
Diabetes mellitus type I*	1 (5.9%)	0	0.356
Diabetes mellitus type II*	3 (17.6%)	3 (21.4%)	0.791
Hepatic comorbidity*	2 (11.8%)	0	0.185
Pulmonary comorbidity*	2 (11.8%)	1 (7.1%)	0.665
Chronic kidney failure*	3 (17.6%)	3 (21.4%)	0.791
**Hospital stay**			
Length of stay in ICU°	16 (4 – 23)	17 (7 – 25)	0.468
Length of stay in hospital°	17 (7 – 37)	24 (11 – 61)	0.297
Bacterial coinfection during stay*	8 (47.1%)	9 (64.3%)	0.337
Fungal Coinfection during stay*	11 (64.7%)	9 (64.3%)	0.981
ICU mortality*	8 (47.1%)	0	0.003
Hospital mortality*	8 (47.1%)	1 (7.1%)	0.015

* n (%); ° median (IQR)

BMI: Body mass index; HBA1c%: glycated hemoglobin; SOFA: sequential organ failure assessment; SAPS III: simplified acute physiology score; COPD: chronic obstructive pulmonary disease; ICU: intensive care unit.

ESM Table S6: Comparison of therapeutic interventions and medication restricted to patients with respiratory focus

		Bacterial (n = 17)	Viral (n = 14)	p
IMV*		12 (70.6%)	12 (85.7%)	0.316
Vasopressors*		17 (100.0%)	12 (85.7%)	0.107
AKI*		12 (70.6%)	8 (57.1%)	0.436
	KDIGO stage 1*	2 (16.7%)	1 (12.5%)	
	KDIGO stage 2*	5 (41.7%)	3 (37.5%)	0.928
	KDIGO stage 3*	5 (41.7%)	4 (50.0%)	
CVVH*		4 (23.5%)	5 (35.7%)	0.457
ECMO*		1 (5.9%)	1 (7.1%)	0.887
IMV days°		16 (8 – 21)	12 (6 – 32)	0.977
NIV days°		3 (1 – 5)	4 (2 – 6)	0.610
NHF days°		2 (1 – 4)	3 (2 – 7)	0.230
CVVH days°		13 (9 – 16)	7 (4 – 19)	0.730
ECMO days°		11 (11 – 11)	5 (5 – 5)	1.000
Use of antibiotics		17 (100.0%)	14 (100.0%)	-
Use of antiviral drugs		0	5 (35.7%)	< 0.001
Methylprednisolone*		2 (11.8%)	2 (14.3%)	0.835
Dexamethasone*		1 (5.9%)	7 (50.0%)	0.005
Hydrocortisone*		15 (88.2%)	3 (21.4%)	< 0.001
Other corticosteroids*		3 (17.6%)	0	0.098
No corticosteroid administration*		2 (11.8%)	1 (7.1%)	0.665
Methylprednisolone days°		10 (1 – 19)	7 (5 – 8)	1.000
Dexamethasone days°		1 (1 – 1)	10 (10 – 20)	0.250
Hydrocortisone days°		8 (4 – 9)	3 (2 – 7)	0.130
Other corticosteroid days°		4 (1 – 6)	-	-
Cumulative dose of Methylprednisolone (mg)°		662 (300 – 1024)	820 (320 – 1320)	1.000
Cumulative dose of Dexamethasone (mg)°		13 (13 – 13)	60 (60 – 198)	0.250
Cumulative dose of Hydrocortisone (mg)°		1069 (550 – 1350)	250 (200 – 1250)	0.164

* n (%); ° median (IQR)

IMV: invasive mechanical ventilation; AKI: acute kidney injury; KDIGO: Kidney Disease: Improving Global Outcomes; RRT: renal replacement therapy; ECMO: extracorporeal membrane oxygenation; NIV: non-invasive ventilation; NHF: nasal high flow; mg: milligram.

ESM Figure S1: Course of inflammatory markers in the subgroup with a respiratory focus

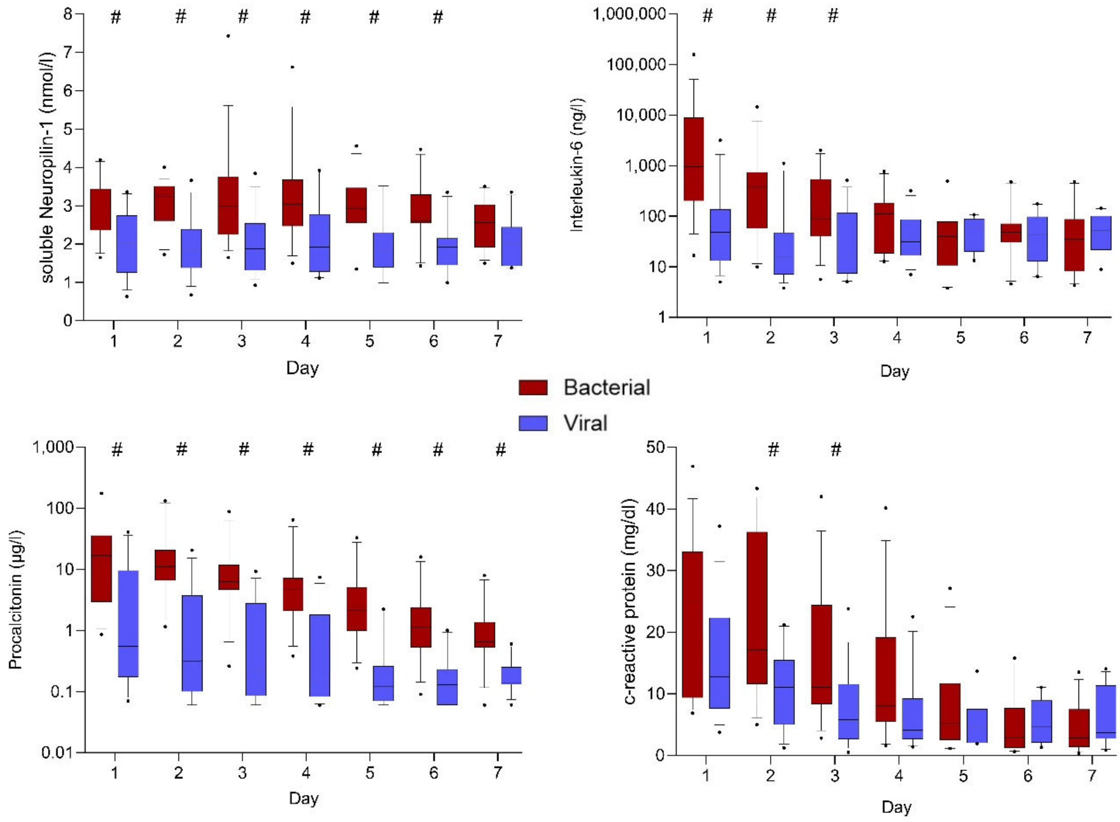

#: indicates statistically significant differences between groups ($p < 0.05$)
